# Glycemic dispersion: a new index for screening high glycemic variability

**DOI:** 10.1186/s13098-023-01077-y

**Published:** 2023-05-09

**Authors:** Rui Shi, Lei Feng, Yan-Mei Liu, Wen-Bo Xu, Bei-Bei Luo, Ling-Tong Tang, Qian-Ye Bi, Hui-Ying Cao

**Affiliations:** 1 Department of Medical Laboratory, Sixth Affiliated Hospital of Kunming Medical University, Yunnan, China; 2 Department of Laboratory Medicine, The Sixth Affiliated Hospital of Guangzhou Medical University, Qingyuan People’s Hospital, Qingyuan, People’s Republic of China; 3 Center Blood Station of Yuxi, Yuxi, China

**Keywords:** Diabetes, Glycemic variability, HbA1c, Glycemic dispersion index

## Abstract

**Objective:**

For patients with diabetes, high-frequency and -amplitude glycemic variability may be more harmful than continuous hyperglycemia; however, there is still a lack of screening indicators that can quickly and easily assess the level of glycemic variability. The aim of this study was to investigate whether the glycemic dispersion index is effective for screening high glycemic variability.

**Methods:**

A total of 170 diabetes patients hospitalized in the Sixth Affiliated Hospital of Kunming Medical University were included in this study. After admission, the fasting plasma glucose, 2-hour postprandial plasma glucose, and glycosylated hemoglobin A1c were measured. The peripheral capillary blood glucose was measured seven times in 24 h, before and after each of three meals and before bedtime. The standard deviation of the seven peripheral blood glucose values was calculated, and a standard deviation of > 2.0 was used as the threshold of high glycemic variability. The glycemic dispersion index was calculated and its diagnostic efficacy for high glycemic variability was determined by the Mann–Whitney U test, receiver operating characteristic (ROC) curve and, Pearson correlation analysis.

**Results:**

The glycemic dispersion index of patients with high glycemic variability was significantly higher than that of those with low glycemic variability (*p* < 0.01). The best cutoff value of the glycemic dispersion index for screening high glycemic variability was 4.21. The area under the curve (AUC) was 0.901 (95% CI: 0.856–0.945) and had a sensitivity of 0.781 and specificity of 0.905. It was correlated with the standard deviation of blood glucose values (*r* = 0.813, *p* < 0.01).

**Conclusions:**

The glycemic dispersion index had good sensitivity and specificity for screening high glycemic variability. It was significantly associated with the standard deviation of blood glucose concentration and is simple and easy to calculate. It was an effective screening indicator of high glycemic variability.

**Supplementary Information:**

The online version contains supplementary material available at 10.1186/s13098-023-01077-y.

## Introduction

Chronic complications of diabetes cause disabilities and increase mortality [1]. Strict control of blood glucose and delaying the occurrence and development of chronic complications are decrease the risk of adverse consequences of diabetes. However, the occurrence and development of diabetes complications may be related not only to persistent hyperglycemia but also to cell and tissue damage from high glycemic variability [2]. High-frequency and high amplitude glycemic variability may be more harmful than constant hyperglycemia [3], and high glycemic variability has been reported to be a reason for the continued high incidence of complications when blood glucose reaches the standard [4]. The 2017 Expert Consensus on the Management of Blood Glucose Fluctuation in Diabetes Patients recommended that ideal glycemic control for diabetes patients not only meet the glycosylated hemoglobin (HbA1c) goal, but also reduce the range of blood glucose fluctuations [5]. International consensus guidelines also recommend monitoring and evaluation of glycemic variability [5–7]. Continuous glucose monitoring (CGM) and self-monitoring of blood glucose (SMBG) are accepted as ways to monitor glycemic variability. However, complicated operation, high price, complex calculation of evaluation indicators, and difficult clinical interpretation have limited the use and compliance of CGM and SMBG by diabetes patients [7, 8]. Most diabetic patients find it difficult to achieve refined glycemic management. Therefore, developing an index that can be used in the clinic to rapidly assess glycemic variability for the diabetic population is needed. Previous studies found that 1,5-anhydroglucitol (*r*² = 0.77, *p* = 0.03) and the ratio of glycosylated albumin (GA) to HbA1c (β = 0.061, *p* = 0.004), both were independently and positively correlated with the Mean Amplitude of Glycemic Excursions [9, 10]. However, the sensitivity and specificity of GA/HbA1c and 1,5-anhydroglucitol for use as indicators to screen glycemic variability has not been investigated. Chinese guidelines recommend 1,5-anhydroglucitol as an indicator of postprandial blood glucose fluctuation [11], but an indicator of glycemic variability is lacking. This study evaluated a novel indicator for screening glycemic variability The glycemic dispersion index (GDI) is calculated by integrating the fasting plasma glucose (FPG), 2 h postprandial plasma glucose (2hPG) and mean blood glucose of diabetes patients. This cross-sectional study used blood glucose data obtained from 170 diabetes patients to explore whether the GDI was correlated with the standard deviation of the blood glucose concentration in diabetes patients. We estimated the sensitivity and selectivity of GDI to screen for high glycemic variability.

## Research design and methods

### Study design

A total of 170 diabetes patients and hospitalized in the Department of Endocrinology of the Sixth Affiliated Hospital of Kunming Medical University between January and December 2021 were included. All met the 2018 diabetes diagnostic and typing criteria of the American Diabetes Association in 2018 [12]. Patients with incomplete blood glucose concentration values, who changed the hypoglycemic treatment plan 1 month before admission, with hypoproteinemia and anemia, with acute infection, serious organic diseases, acute diabetes complications, or other diseases that could lead to stress hyperglycemia, or were using glucocorticoids, thyroid hormones, β-adrenergic agonists or other drugs that could affect blood glucose concentration were excluded.

### Inspection index

Patient characteristics included sex, age, and body mass index (BMI). Laboratory values, including FPG, 2hPG, HbA1c, GA, alanine aminotransferase (ALT), creatinine (CREA), hemoglobin (HGB), and peripheral blood glucose were collected within 24 h after admission. Peripheral blood glucose was measured seven times in 24 h, before and after meals, and before going to bed before changing the hypoglycemic treatment plan.

### Test methods and instruments

In the morning of the second day after admission, and after fasting for more than 8 h, 5 mL and 2 mL of venous blood were collected in vacuum tubes containing separation gel coagulant and EDTA-K2, respectively. The tubes were inverted eight times for mixing, and FPG, ALT, CREA and GA were assayed using an autoanalyzer (Roche Cobas C701) using the hexokinase, rate, sarcosine oxidase, and colorimetric methods, respectively. HGB and HbA1c% were assayed by immunoturbidimetry with an autoanalyzer (Cobas c502; Roche, Switzerland). The HbA1c% test was certified by the National Glycohemoglobin Standardization Program. After collecting fasting venous blood, 75 g anhydrous glucose was dissolved in 250 mL water and given orally. Venous blood was collected for glucose assays after 1, 2, and 3 h. After admission, bedside blood glucose meters (Yuyue 582) monitored blood glucose in peripheral capillaries using the oxidase method before and after three meals and before sleep. The SD of seven peripheral blood glucose values was calculated. The hypoglycemic treatment plan of the patient remained the same as before admission.

### GDI formula derivation

**Fig. 1:**
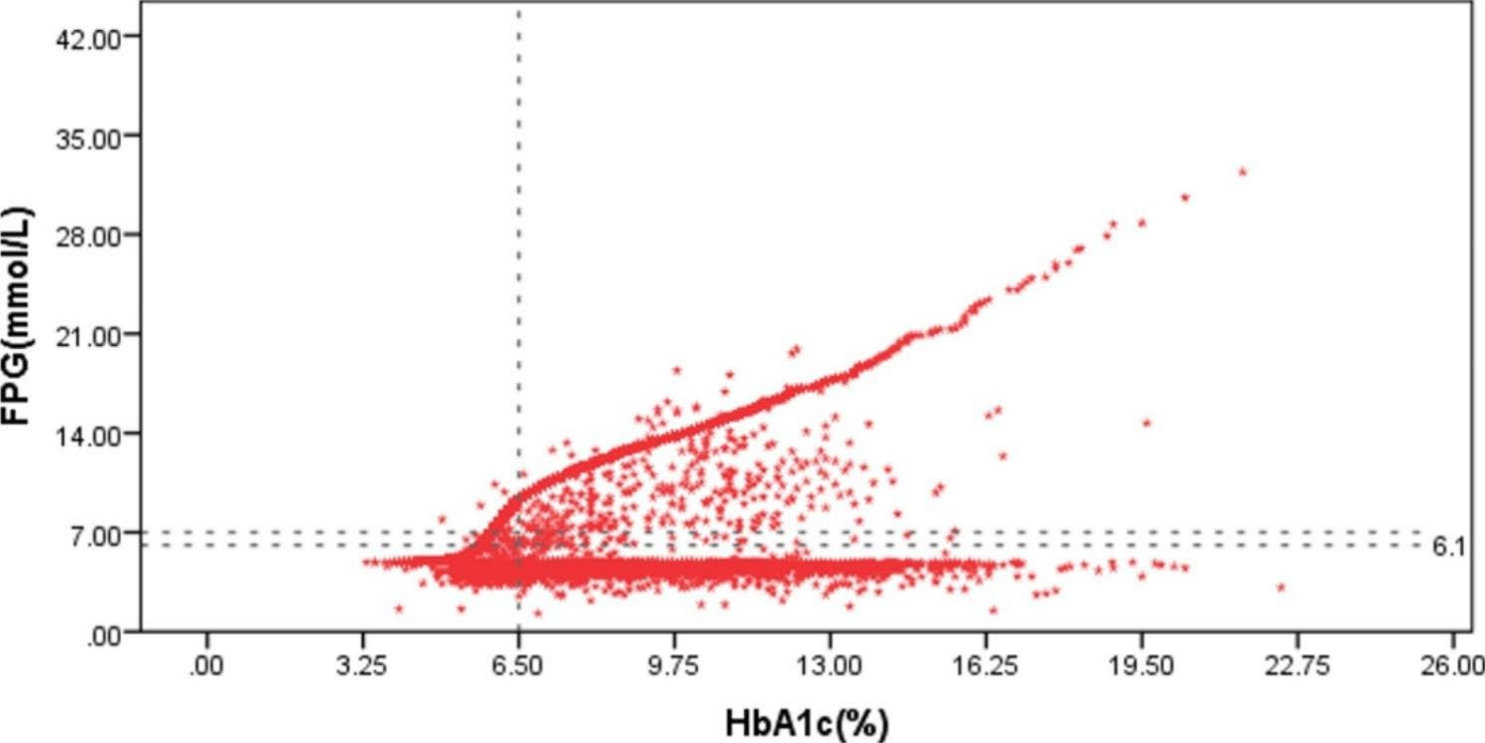
Scatter plot of FPG and HbA1c% values in 15,312 diabetes patients

We previously reported that in the general population in China, increases of FPG and HbA1c were positively correlated [13]. However, in 15,312 patients with type 2 diabetes, a large proportion of patients had poor correlation between FPG and HbA1c%, with normal FPG values and an HbA1c% above the normal range (Fig. 1).

Poor glycemic control in those patients led to fluctuation of blood glucose that was most likely reflected by an increase of the HbA1c%. The relationship between HbA1c% and FPG might be useful as an indicator of glycemic variability. A Chinese Expert Consensus concluded that the increase in postprandial blood glucose and hypoglycemia constitute the daily fluctuation of blood glucose fluctuation [14], Considering the relationship between HbA1c% and FPG, while ignoring postprandial blood glucose, might not accurately reflect glycemic variability. Thus, 2hPG, which is a well-known, widely-used measure in the clinical treatment of T2DM patients was included in the formula used to calculate GDI. International and Chinese guidelines and an expert consensus recommend using the SD of the blood glucose concentration for evaluating glycemic variability [5–7]. The 2017 Expert Consensus on Blood Glucose Fluctuation Management recommended an SD ≥ 2.0 as the threshold of high glycemic variability. Seven blood glucose measurements are recommended, before and after meals and before going to bed [5]. The GDI is derived from the formula used to calculate the SD of blood glucose concentration, i.e. the reference formula:

$SD = \sqrt{\frac{\sum _{i=1}^{n}({x}_{i}-AG{)}^{2}}{n-1}}$, where *x =* blood glucose concentration, AG = mean blood glucose concentration, and *n =* total number of blood glucose values.

As only FPG and 2hPG are included in the GDI formula, ${x}_{1}=FPG?{x}_{2}=2?PG$, and *n* = 2. HbA1c% can be used to estimate the average glucose (eAG) in the previous 2–3 months [15], which is calculated as eAGmmol/l = 1.59 × HbA1c%–2.59, To simplify patient evaluation we used eAG instead of AG. The GDI was calculated as:

$$\begin{array}{c}GDI = \sqrt {\frac{{{{\left( {FPG - eAG} \right)}^2} + {{\left( {2hPG - eAG} \right)}^2}}}{{2 - 1}}} \\\downarrow \\GDI = \sqrt {{{\left( {FPG - eAG} \right)}^2} + {{\left( {2hPG - eAG} \right)}^2}} \end{array}$$

### Statistical analysis

SPSS Statistical Software 24.0 was used for the statistical analysis. The Kolmogorov-Smirnov test showed that BIM, 1hPG, and 2hPG were normally distributed and that AGE, HGB, FPG, HbA1c%, GA, ALT, and CREA were not. The unpaired *t*-test was used to compare between-group differences of normally distributed data. The Mann–Whitney U test was used to compare data that were not normally distributed. Results with a normal distribution were reported as means ± SD. Those that were not normally distributed were reported as medians and first and third quartile (Q1, Q3), Pearson correlation coefficients were used to analyze the significance of correlations between variables. Receiver operating curve (ROC) curves and areas under the curve (AUC) calculated with 95% confidence intervals (CIs) determine the sensitivity and selectivity of study variables for screening high glycemic variability. The DeLong method was used to compare AUCs. The significance level was *p* < 0.05.

## Results

### Baseline characteristics and comparison of patients with high and low glycemic variability

**Fig. 2:**
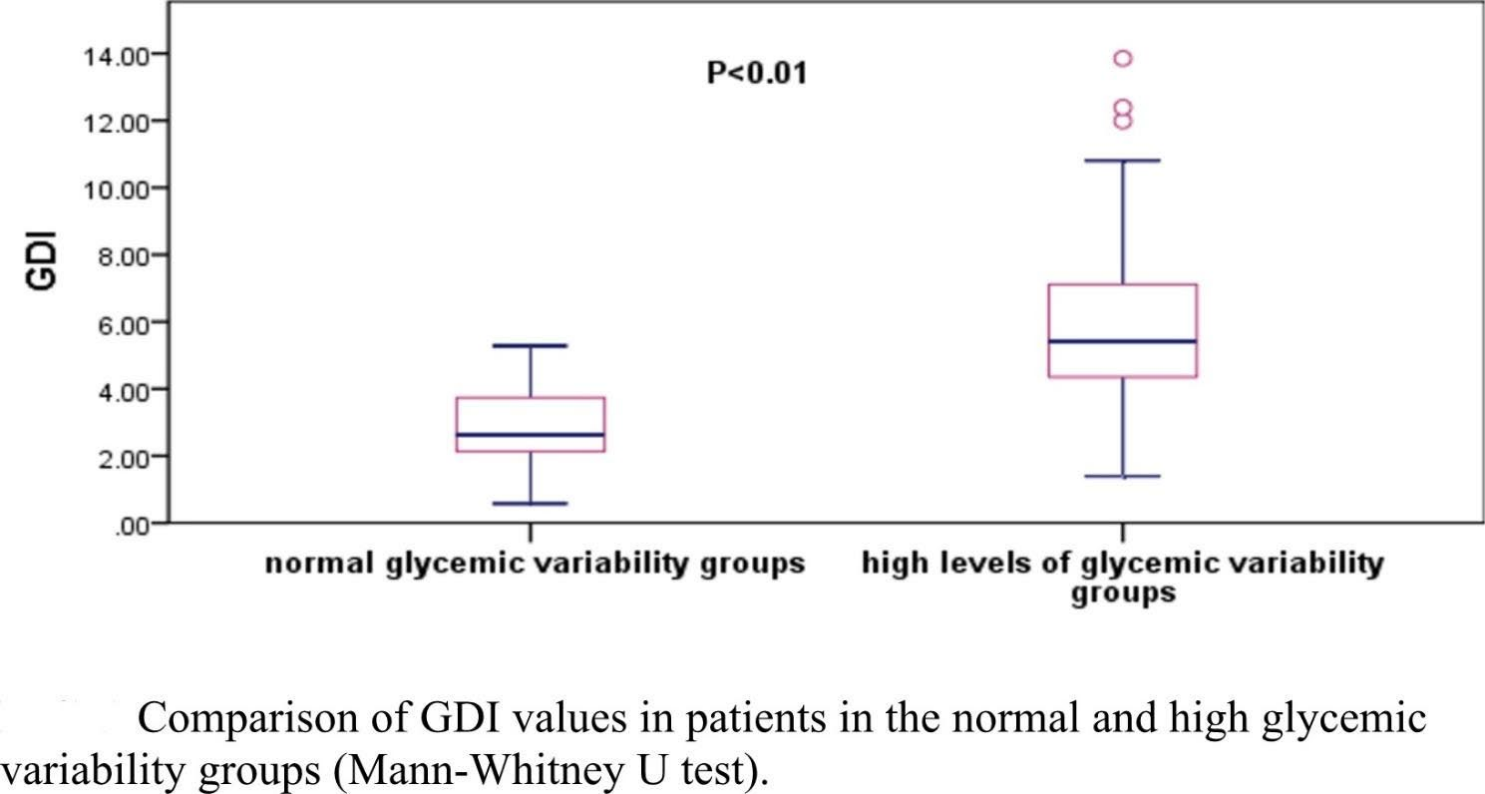
Comparison of GDI values in patients in the normal and high glycemic variability groups (Mann-Whitney U test)

Seventy-four patients, 46 men and 28 women, had normal glycemic variability and 96, 53 men and 43 women, had high variability. Differences in AGE, HGB, FPG, CREA, and BMI in the two groups were not significant (p > 0.05). HbA1c%, GDI, 1hPG, and 2hPG were significantly lower in the normal glycemic variability group than in the high variability group (p < 0.05), ALT was higher in the normal glycemic variability group than in the high variability group (p < 0.05, Table 1). The median GDI in the high glycemic variability group was 5.41 (4.30?7.11), which was significantly higher than that in the normal glycemic variability group 2.63 (2.17?3.74, p < 0.001, Fig. 2).

**Table 1:** Comparison of the clinical characteristics in patients with normal and and high glycemic variability

	SD of blood glucose value		*p* value
	≤ 2.0 (n = 74)	> 2.0(n = 96)	
Age (years)	58 (48–67)	59 (50–70)	0.289
HGB (g/dL)	10.67 (10.22–11.92)	11.09 (10.14–12.37)	0.328
FPG (mmol/L)	6.40 (5.54–7.26)	7.06 (5.75–7.94)	0.041
CREA (umol/L)	77 (62–88.5)	77 (64–92)	0.982
HbA1c (%)	6.65 (6.10–7.13)	7.75 (7.10–8.95)	0.000
GDI	2.63 (2.14–3.74)	5.42 (4.35–7.12)	0.000
ALT (u/L)	23.5 (17–37.25)	19.5 (15–28)	0.033
BMI (kg/m2)	24.64 ± 3.31	24.46 ± 3.59	0.727
1hPG (mmol/L)	10.07 ± 2.33	11.88 ± 2.95	0.000
2hPG (mmol/L)	10 ± 2.30	13.56 ± 3.68	0.000

Data are expressed as the mean ± SD or median [Q1, Q3]; HGB, hemoglobin; FPG, fasting plasma glucose; CREA, blood creatinine; HbA1c%, percentage of glycosylated hemoglobin A1c; GDI, glycemic dispersion index; ALT, alanine aminotransferase; BMI, body mass index; 1hPG, 1-h postprandial plasma glucose; 2hPG, 2-h postprandial plasma glucose

### Effectiveness of GDI, |2hPG-FPG|, HbA1c%, and GA/HbA1c for screening high glycemic variability

**Fig. 3:**
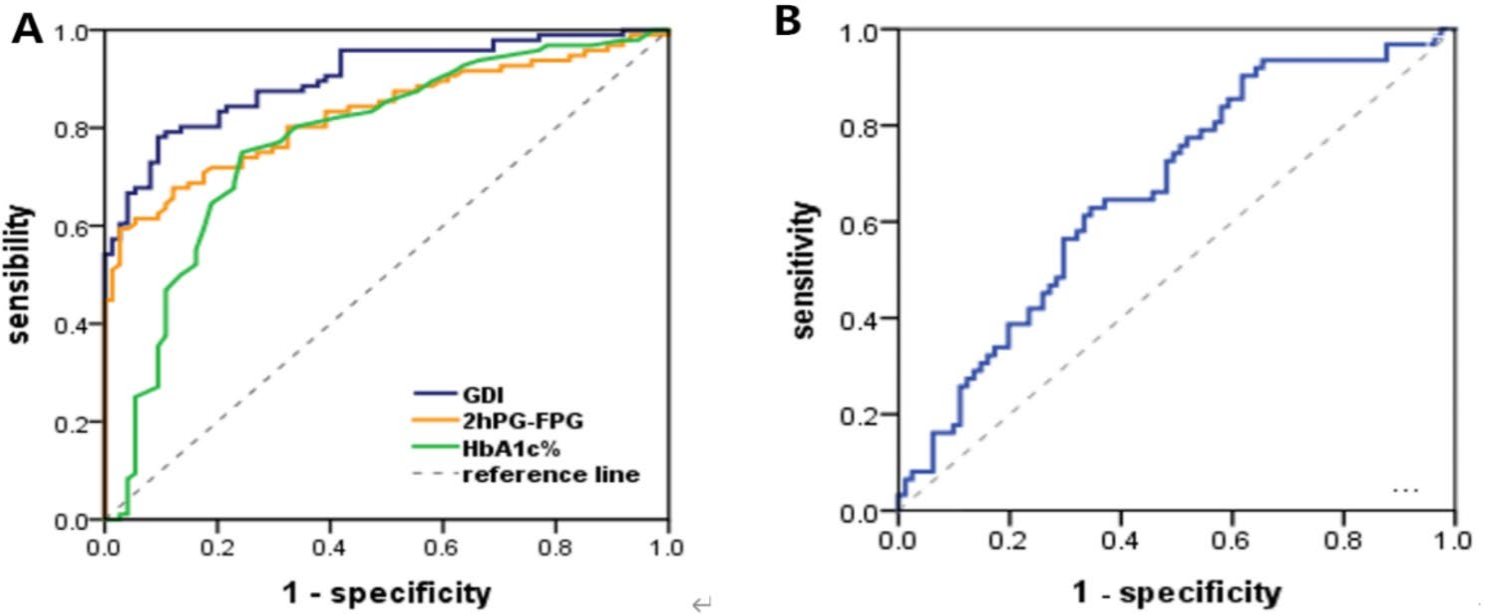
**A** ROC curves of GDI, |2hPG—FPG|, and HbA1c% for screening high levels of glycemic variability. **B** ROC curve of GA/HbA1c for excluding high levels of glycemic variability

ROC curve analysis found the best cutoff values for screening glycemic variability were 4.21 for the GDI, 5.89 for |2hPG?FPG|, and 7.15 for HbA1c%. The corresponding AUCs were 0.901 (95% CI: 0.856?0.945), 0.827 (95% CI: 0.765?0.888), and 0.774 (95% CI: 0.701?0.848). The sensitivities were 0.781, 0.594, and 0.75, and the specificities were 0.905, 0.973, and 0.757 respectively. The best cutoff value of GA/HbA1c to exclude high glycemic variability was 0.42, the AUC was 0.66 (95% CI: 0.689?0.878), the sensitivity was 0.903, and the specificity was 0.383 (Table 2). The AUC of GDI was significantly different from the| 2hPG?FPG |, HbA1c% and GA/HbA1c (p < 0.001; Fig. 3).

**Table 2:** Sensitivity, specificity, positive likelihood ratio, negative likelihood ratio, and AUC of GDI, |2hPG-FPG|, HbA1c%, and GA/HbA1c for screening high glycemic variability

	Cut-off	Se	Sp	PLR	NLR	AUC (%)
GDI	4.21	0.781	0.905	8.22	0.24	0.901
**|**2hPG-FPG**|**	5.89	0.594	0.973	22.00	0.42	0.828
HbA1c%	7.15	0.75	0.757	3.09	0.33	0.774
GA/HbA_1c_	0.42	0.903	0.383	1.56	0.25	0.666

GDI, glycemic dispersion index; |2hPG-FPG|, absolute value of difference between 2-h postprandial plasma glucose and fasting plasma glucose; HbA1c%, percentage of hemoglobin A1c; GA/HbA_1c_, ratio of glycosylated albumin-to-glycosylated hemoglobinA1c; Se, sensitivity; Sp, specificity; PLR, positive likelihood ratio; NLR, negative likelihood ratio; AUC, area under the ROC curve

### Correlation of GDI, |2hPG–FPG|, HbA1c%, and GA/HbA1c with the SD of blood glucose concentration

 The correlations of GDI (*r* = 0.813), |2hPG?FPG| (*r* = 0.736), HbA1c% (*r* = 0.466), and GA/HbA1c (*r* = −0.248) and the blood glucose SD significant (all p < 0.01, Table 3).

**Table 3:** Correlation of GDI, |2hPG-FPG|, HbA1c%, and GA/HbA1c with the SD of the blood glucose concentration

	r	p
GDI	0.813	< 0.01
**|**2hPG-FPG**|**	0.736	< 0.01
HbA1c%	0.466	< 0.01
GA/HbA1c	−0.248	< 0.01

GDI, glycemic dispersion index; |2hPG-FPG|, absolute value of difference between 2-h postprandial plasma glucose and fasting plasma glucose; HbA1c%, percentage of hemoglobin A1c; GA/HbA1c, ratio of glycosylated albumin-to-glycosylated hemoglobinA1c; r, Pearson correlation coefficient

## Discussion

The International Diabetes Federation Global Diabetes Map tenth edition, 2021 estimated that there were 537 million diabetes patients between 20 and 79 years of age worldwide, with about 6.7 million deaths of adults from diabetes or its complications [16]. The results of studies conducted in the last 20 years have confirmed that high glycemic variability increases oxidative stress, induces inflammatory reactions, and promotes endothelial-cell damage ion diabetes patients [2]. It is also an independently associated with increased risk of macrovascular and microvascular complications and neurological dysfunction [17–19]. If glycemic variability is not well monitored and controlled, the occurrence and progression of diabetes complications cannot be delayed even if HbA1c is well controlled [4]. Effective monitoring of glycemic variability cannot be ignored, but the limitations of CGM and SMBG result in low compliance and inadequate monitoring. The GDI is a simple, convenient screening index for high glycemic variability. To explore the screening effectiveness of GDI, the study found that the GDI value was higher in patients with high glycemic variability than it was in patients with normal glycemic variability. ROC curve analysis showed that AUC of the GDI as a screening tool was significantly higher than the AUCs of |2hPG-FPG|, HbA1c%, and GA/HbA1c. Although the specificity (0.973) and positive likelihood ratio (21.59) of |2hPG—FPG| were higher than those of GDI, its sensitivity (0.594) was lower. As only |2hPG-FPG| was measured, which are affected by diet, mood, drugs and other factors, it is not suitable for screening. The relationship of GA/HbA1c and glycemic variability was studies in 143 diabetes patients in this study, 62 with normal glycemic variability and 81 with high glycemic variability. The sensitivity of GA/HbA1c to exclude high glycemic variability was high (0.903), but its specificity (0.383) and positive likelihood ratio (1.56) were too low to be suitable from screening. The variability of HbA1c is significantly associated with microvascular and macrovascular complications of diabetes [20, 21]. However, the long cycle of HbA1c variability monitoring is not conducive to timely monitoring of glycemic variability, The screening efficacy of HbA1c% for high glycemic variability, the sensitivity (0.75) and specificity (0.757) of HbA1c% were significantly lower than the GDI in the current study.

GDI had high specificity (0.905) and sensitivity (0781) and was positively correlated with the SD of blood glucose values (*r* = 0.813, *p* < 0.01). Its good screening efficacy is attributable to the evaluation factors included in the calculation of the model. It not only includes the low (FPG) and high (2hPG) blood glucose concentrations but also considers that because of the daily differences in blood glucose values, the glycemic variability in a one day may not accurately reflect glycemic variability over a longer period of time, Therefore, the GDI formula includes eAG, which reflects the mean blood glucose concentration in the previous 2–3 months, which buffers daily differences, accidental hyperglycemia, and missed diagnosis of hyperglycemia and hypoglycemia. Taking the original glycemic data of the three cases included in this study as examples for explanation (see Supplementary Information).

The SD of the blood glucose value reflects mainly depends on the difference between each blood glucose value and the mean level of blood glucose, the greater the difference, the greater the dispersion between each blood glucose value and the mean level of blood glucose, which means the greater the variability of glycemic. Therefore, determining the mean level of blood glucose levels is a key step in evaluating glycemic variability. Without frequent blood glucose monitoring, HbA1c is the best indicator to reflect the mean level of blood glucose of patients, and it is not affected by lifestyle changes such as short-term diet and exercise [6]. The formula for converting HbA1c% to eAG in GDI has been recommended by the 2020 Chinese Guidelines for the Prevention and Treatment of Type 2 diabetes [11]. When FPG and 2hPG accurately reflect the low and high blood glucose concentrations, the GDI formula calculates the sum of the differences between FPG, 2hPG and eAG, to measure the maximum dispersion of blood glucose value to evaluate glycemic variability.

The inclusion of HbA1c% increased the sensitivity of GDI to screen high glycemic variability. HbA1c production increases with the increase in blood glucose concentration and of the duration of the increase [6]. If FPG and 2hPG do not accurately reflect the patient’s low and high blood glucose concentration, there is hidden hypoglycemia or hyperglycemia. The HbA1c% decreased or increased with glucose concentration and duration of sub-clinical hypoglycemia or hyperglycemia. If difference between FPG or 2hPG and eAG increases, GDI increases. GDI evaluates not only glycemic variability but also shows whether patients have hidden hypoglycemia or hidden hyperglycemia.

Secondly, GDI was effective for screening high glycemic variability and its calculation was simpler than that of traditional indices such as SD and coefficient of variation. More important, it is only necessary to collect whole blood on an empty stomach and 2 h after a meal to monitor GDI, GDI monitoring only requires twice of invasive blood collection, Compared with SMBG monitoring, the number of blood collection is reduced by five times. The operation is simpler, which improves the compliance of patients. Compared with CGM, it greatly reduces the economic burden and pressure of patients to comply.

### Population not applicable to GDI

Patients who changed hypoglycemic treatment within 1 month before testing HbA1c. Research has shown that the mean blood glucose in the 3 months before HbA1c measurement has an impact on HbA1c levels of 50%, 30%, and 20%, respectively [22]. The eAG used in the GDI formula is converted from HbA1c relatively accurately reflected the mean blood glucose of the patient in the month before testing; however, if diabetes patients have changed the hypoglycemic treatment within 1 month before HbA1c measurement, the detected FBG and 2hPG may undergo significant changes under the influence of drugs, thus losing the correlation with HbA1c, and resulting in the calculated GDI not reflecting the true level of glycemic variability.

T1DM patients. As is well-known, compared to T2DM, T1DM patients have more severe blood glucose variability and with frequent hypoglycemic episodes and a severe form of the dawn phenomenon [23]. For T1DM patients, a severe dawn phenomenon, the FBG value cannot accurately reflect a low blood glucose concentration within 24 h; even frequent hypoglycemia may be far below the FBG value. Due to severe damage to the function of pancreatic B cells, the peak secretion of C-peptide is severely delayed, the 3 h postprandial plasma glucose or even 4 h can still be higher than the 2hPG, and 2hPG levels cannot reflect the high glucose concentration within 24 h. Therefore, GDI is not suitable for T1DM patients.

In summary, GDI was effective for screening glycemic variability, is mor convenient than CGM and SMBG, it is simple to operate, low in cost, and has high clinical applicability, Patients with normal glycemic variability screened by GDI can avoid unnecessary blood glucose monitoring methods such as CGM or SMBG, reduce the financial burden of patients and alleviate anxiety. Patients with `high glycemic variability screened by GDI can perform SMBG or CGM monitoring to obtain more detailed information on blood glucose fluctuations. Therefore, monitoring GDI can expand the population being monitored for glycemic variability, provide early assessment and warning of the risk of occurrence, development of diabetes complications. It can be a target of primary and secondary prevention, guide clinical diagnosis and treatment, provide personalized hypoglycemic treatment programs, and facilitate the development of precision medicine for diabetes.

This study has limitations. First, it excluded patients with hypoproteinemia, anemia, acute infection, severe organic disease, acute diabetes complications, and recent use of glucocorticoids, thyroid hormone, β-adrenergic agonists, and other drugs that affect blood glucose levels. Consequently, GDI may not be useful in such patients. Additionally, to ensure that the study results accurately describe the daily glycemic variability, we monitored blood glucose concentration of patients within 24 h of admission and before making any changes in treatment. As few patients had complete blood glucose data including all seven required assays on the first day, leading to the small sample size included in the study. A large prospective study is required to verify the study results.

## Electronic supplementary material

Below is the link to the electronic supplementary material.

Supplementary Material 1

## Data Availability

The datasets used and/or analyzed during the current study are available from. the corresponding author on reasonable request.
