# Supplementary material for: Glycemic dispersion: a new index for screening high glycemic variability
Source: Diabetol Metab Syndr. 2023 May 9;15:95. doi: 10.1186/s13098-023-01077-y (PMC10169464; doi:10.1186/s13098-023-01077-y)
Supplement: Supplementary file 1 — Supplementary Material 1 [file 13098_2023_1077_MOESM1_ESM.doc]

**SUPPLEMENTARY INFORMATION**

**Glycemic Dispersion: A New Index for Screening** **High Glycemic Variability**

Rui Shi ; Lei Feng ; Yan-Mei Liu; Wen-Bo Xu ; Bei-Bei Luo ; Ling-Tong Tang; Qian-Ye Bi ; Hui-Ying Cao

Details of three cases included in this study（See Supplementary Data 1 for original data）

The FPG of patient No. 48 was 5.92 mmol/L and 2hPG was 11.08 mmol/L the difference between FPG and 2hPG was 5.16 mmol/L, which exceeded the maximal range of <4.4 mmol/L specified by the Consensus of Chinese Experts in 2017.^5^ However, the actual glycemic variability of the patient was normal. The SD of blood glucose values was 1.77 mmol/L, the GDI was 3.74, which was less than the cutoff value of 4.21 mmol/L, eAG=7.90, indicating that the patient's increase of 2hPG increase may have been incidental and little impact on the overall glycemic variability. Under such circumstances, the patient did not need CGM or SMBG monitoring, only continued to observation to see whether there is a significant increase in postprandial blood glucose.

The FPG of the patient No. 136 was 4.83 mmol/L and the 2hPG was 7.41 mmol/L. Blood glucose and glycemic variability seemed to be well controlled, but the SD of blood glucose values was 3.18, the GDI was 9.45, which was greater than the cutoff value of 4.21, the eAG was 12.67, which indicated high glycemic variability, with a large increase in glucose concentration. CGM or SMBG monitoring should be performed to determine the time of the increase in glucose concentration, observe the variability, and investigate the reasons, with timely intervention to adjust living habits and drug intervention.

The FPG of the patient No. 151 was 11.41 mmol/L and the 2hPG was 15.03 mmol/L. The overall daily blood glucose concentration appeared to increase significantly, and although the difference was relatively small, glycemic variability was high, with an SD of 3.64. The GDI was 7.73, which was higher than the cutoff value of 4.21., indicating high glycemic variability and a hypoglycemic time point. In addition to hypoglycemic treatment, such patients need blood glucose monitoring to determine when hypoglycemia occurs, such as before bed, or early morning, to avoid serious consequences caused by covert hypoglycemia, which is particularly important for elderly patients with diabetes.
